# Motion matters: visual perception and motor functioning in children and youths with spastic cerebral palsy

**DOI:** 10.3389/fneur.2026.1921002

**Published:** 2026-09-25

**Authors:** Annika Ericson, Kerstin Hellgren, Mariagrazia Benassi, Roberto Bolzani, Cecilia Lidbeck

**Affiliations:** 1 Division of Paediatric Neurology, Department of Women's and Children's Health, Karolinska Institutet, Stockholm, Sweden; 2 Astrid Lindgren Children's Hospital, Karolinska University Hospital, Stockholm, Sweden; 3 Division of Eye and Vision, Department of Clinical Neuroscience, Karolinska Institutet, Stockholm, Sweden; 4 Department of Psychology, University of Bologna, Bologna, Italy

**Keywords:** cerebral palsy, cerebral visual impairment, motion perception, motor functioning, visual perception

## Abstract

**Background:**

Vision plays a crucial role in guiding and anticipating movements. In individuals with cerebral palsy (CP), a condition characterized by disorders of movement and posture attributed to disturbances of the developing brain, Cerebral Visual Impairments (CVI) are common. CVI involves difficulties with organizing and interpreting visual information. Gait alterations and activity limitations in children with CP such as reduced; walking speed, step length, and motor skill performance, may be over-attributed to the motor and musculoskeletal disorders. Knowledge of how visual perception influence motor functioning in individuals with CP is sparse. The aim of this study was to investigate global motion perception, the ability to interpret the coherent direction of visual motion, and reported features of CVI in children and youths (C&Y) with spastic CP at different levels of motor functioning.

**Methods:**

C&Y aged 8–20 (mean age 14:4) years, CP; 37 (gross motor functioning classification system, GMFCS, I; 28, II-III; 9) and typically developing (TD); 36, performed a computer-based Visual Motion Coherence Test (VMCT) evaluating global motion perception at three velocities (score: 0.0–1.0, low scores indicate difficulties). Corrected visual acuity was normal/near normal in all C&Y. The Raven's CPM was used to evaluate fluid intelligence. Parents of C&Y with CP; 67 (GMFCS I; 37, II-IV; 30) and TD; 40, completed a questionnaire capturing features of CVI (CVIQ) (score: 0–156, high scores indicate CVI).

**Results:**

Global mean (SE) VMCT score, controlled for age and fluid intelligence, was lower in the CP-group than the TD-group; 0.64 (0.03) vs. 0.70 (0.01), (*p* = 0.04), and lower at high and low velocities vs. medium, most pronounced at GMFCS II-III. The median CVIQ score was higher in the CP-group than the TD-group; 28.0 vs. 4.5 (*p* < 0.001), and in the GMFCS II-IV-group (*n* = 30) compared to the GMFCS I-group (*n* = 37); 45.5 vs. 24.0 (*p* = 0.003).

**Conclusions:**

A velocity-dependent vulnerability in global motion perception and many reported features of CVI were found in the C&Y with spastic CP, more so in the group with greater limitations in motor functioning. Visual perception should be considered when evaluating functioning and supporting perceptual and motor development in children with spastic CP.

## 1 Background

Vision plays a crucial role in guiding and anticipating movements, making it a factor to consider when evaluating motor performance and development (1). In children, reduced motor skill performance and gait alterations have been related to visual impairments (2, 3). In cerebral palsy (CP), a group of disorders of the development of movement and posture, visual impairments are prevalent and considered a core disorder (4, 5). Despite this, knowledge of how vision influences motor functioning in individuals with CP has been described as limited (6).

With a prevalence of around 2/1,000 live births in developed countries (7), CP is the most common disorder in children causing activity limitations. The activity limitations are often, but not exclusively, related to walking and mobility (4, 7). Cerebral palsy originates from disturbances of the developing brain, and accompanying disturbances such as with sensation, perception, and cognition are prevalent (4), and usually more common in individuals with greater limitations in gross motor functioning (8). Perception can be described as the capacity to incorporate and interpret sensory information from vision and other sensory modalities (4).

The prevalence of ophthalmological abnormalities in children and youths (C&Y) with CP has been reported to vary from 34–100% in a review (9). Commonly occurring ophthalmological abnormalities in the population are refractive errors, strabismus, reduced visual acuity, unstable fixation, and restricted visual fields (9, 10). Moreover, a high prevalence of cerebral visual impairment (CVI), involving difficulties with interpreting visual information, has been reported in children with CP (11). Cerebral visual impairment can be described as “a verifiable visual dysfunction which cannot be attributed to disorders of the anterior visual pathways or any potentially co-occurring ocular impairment” (12). Symptoms of CVI can include difficulties such as with distance and depth estimation, and perceiving details and motions in the environment but may also include ophthalmological abnormalities (13). Detecting CVI can be challenging, and questionnaires are often used to describe visual ability in daily life and capture features of CVI (14–16).

Processing sensory information from vision and the somatosensory system into meaningful information is essential to move and function effectively in the environment (1). Motor functioning in CP can be classified into five levels (I-V) with the Gross Motor Functioning Classification System (GMFCS E&R) where children at GMFCS I, walk without limitations and those at GMFCS II-V show increasing limitations with self-initiated movements (17). Prior research suggests that children with CP rely more on their vision for posture (18–20) and for walking speed (21) compared to typically developing (TD) children, to compensate for somatosensory disturbances, more so in the group of children functioning at GMFCS II-III. Others have reported a sensitivity in analyzing visual stimuli such as moving objects or depth in the close surrounding space in children with spastic CP due to a perceptual disorder (22). The perceptual disorder was described as hindering motor development and impacting the ability to walk and act in a dynamic environment (22).

Visual motion perception involves selecting, organizing, and interpreting visual motion stimuli to anticipate potential collisions, location in depth, and self-motion (23). The ability to detect coherent visual motion, hereafter referred to as global motion perception, is commonly assessed with computer-based tests (24, 25). In children born at risk of abnormal development and TD toddlers, low scores on tests of global motion perception have been associated with low performance on standardized measures of visual motor integration, motor coordination, and gross motor skills (26, 27). Global motion perception is sparsely explored in individuals with CP and little is known about the relationship to motor functioning. In a study on children with unilateral CP, altered global motion perception was reported in comparison to a TD-group (28).

This study aimed to investigate visual perception, focusing on global motion perception, and reported difficulties with vision-related activities in daily life, in C&Y with spastic CP at different levels of motor functioning. The hypotheses were that C&Y with CP would be more sensitive to the velocity of visual motion stimuli and have more reported difficulties with vision-related activities in daily life, more so at higher GMFCS levels, compared to TD C&Y.

## 2 Methods

### 2.1 Participants and procedure

In this observational study, inclusion criteria were entry age of 6–18 years, a verified diagnosis of spastic CP with unilateral (involving limbs on one side of the body) or bilateral distribution (involving limbs on both sides of the body), GMFCS I, II, III or IV, and the ability to follow test instructions. All available participants with CP were recruited in conjunction with scheduled outpatient visits at the neuro pediatric department at the Karolinska University Hospital in Stockholm, Sweden. A convenience sample of TD children at the same ages served as a comparison.

Parents answered a questionnaire designed to capture features of CVI (CVIQ) and grade performance on vision-related activities in daily life (16). Subsequently, all C&Y were invited to perform tests of global motion perception with a computer-based Visual Motion Coherence Test (VMCT) (24, 25, 29, 30), a test of visual acuity, and a test of general analytical ability with the Raven's colored progressive matrices (Raven's CPM) (31) (flow chart in Figure 1).

**Figure 1:**
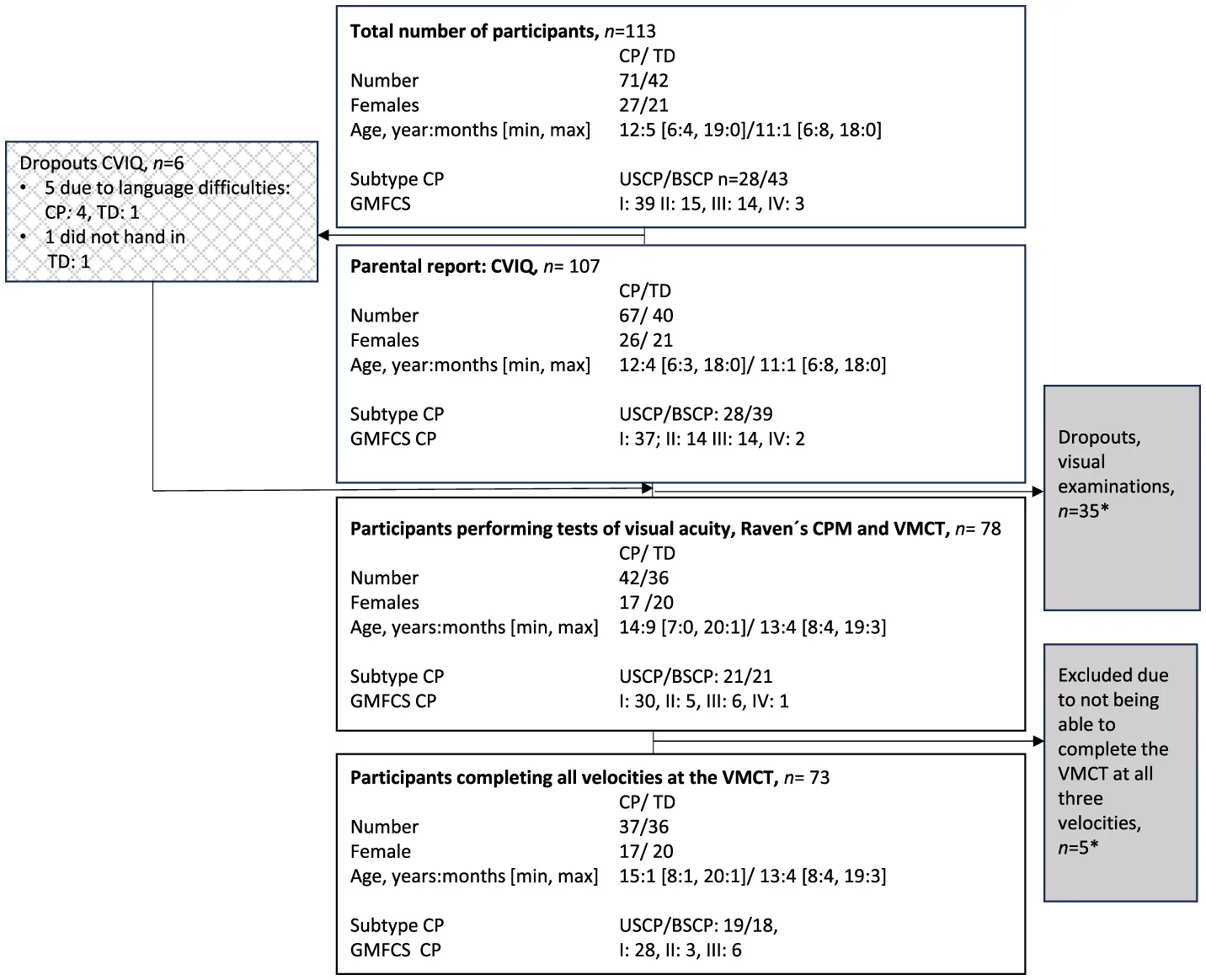
Flow chart with number (*n*) of participants, with cerebral palsy (CP) with unilateral spastic CP (USCP) and bilateral spastic CP (BSCP), at different levels of motor functioning as classified with the Gross Motor Function Classification System (GMFCS) and typically developing (TD) participants, for the respective assessments. The measurements were: a questionnaire for detecting features of Cerebral Visual Impairments (CVIQ), the Visual Motion Coherence Test (VMCT) for evaluating global motion perception, a test of visual acuity, and Raven's colored progressive matrices (Ravens CPM) for measuring fluid intelligence. *, Characteristics in Supplementary Tables, Additional File 1. 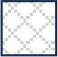 = represents the individuals performing visual tests but not contributing with data to the CVIQ. 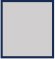 = represents dropouts and excluded individuals.

An orthoptist assessed visual acuity, and a physiotherapist administered the CVIQ, the VMCT, and the Raven's CPM at Astrid Lindgren's Children's Hospital in Sweden. Data was collected from March 2019 to July 2022.

### 2.2 Visual Motion Coherence Test (VMCT)

The VMCT was used to evaluate the accuracy of global motion perception (29, 30). The test is computer-based and examines the ability to detect the coherent motion direction of 150 white high-luminance dots with a brightness of 51.0 candelas ${\text{(cd)/m}}_{.}^{2}$ Each dot subtends an angle of 2.97 arcminutes. On the screen, the dots are packed with 7.6 dots/degree^2^, and they move in a circular frame (5 degrees) on a black background (0.2 cd/m^2^) on a computer screen (in this study size 14”), Figure 2.

**Figure 2:**
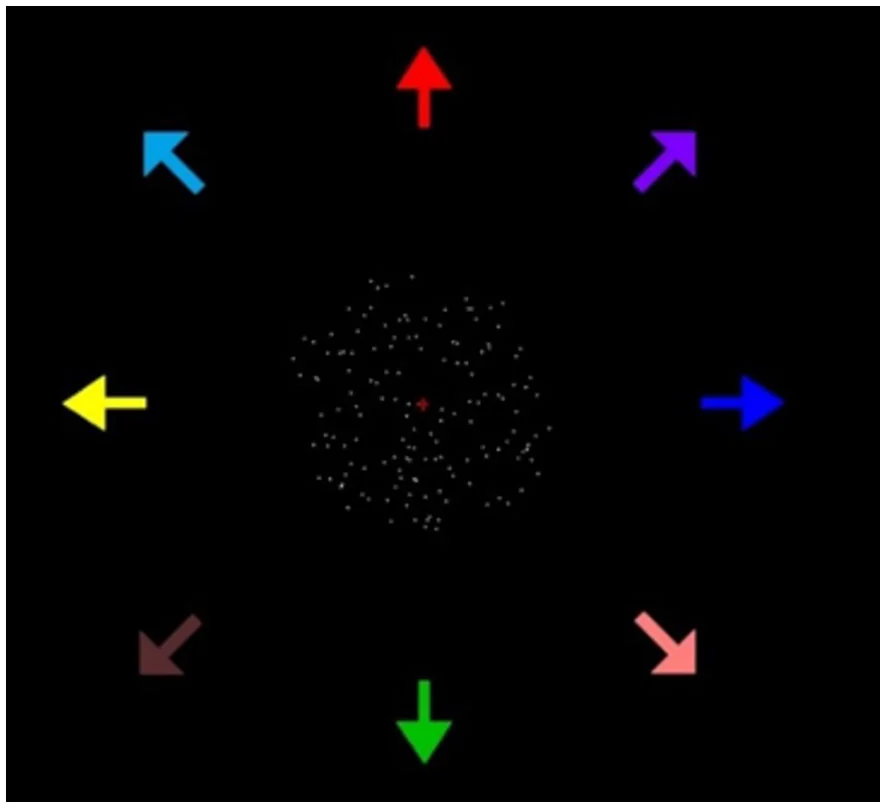
A picture of the Visual Motion Coherence Test (VMCT) evaluating global motion perception as presented on the computer screen. When performing the test, some white high-luminance dots move coherently and others randomly. The red cross in the middle is stationary. The coloured arrows indicate the eight possible coherent motion directions of the high-luminance dots.

Three programs with different velocities; low, moderate and high, were presented in a pseudo-randomized order. The velocity of the stimuli was given by manipulating the temporal frequency of the moving dots while maintaining the same spatial frequency (Table 1).

**Table 1:** Properties for the three velocity programs (low, medium and high velocity) of the Visual Motion Coherence Test (VMCT).

Velocity	Low	Medium	High
Steps per sec	10	20	30
Δx (deg)	0.074	0.074	0.074
Δt (sec)	0.100	0.050	0.033
Vel (deg/sec)	0.739	1.477	2.216

The different speeds are obtained with a constant spatial amplitude (Δx) of each step moved at an increasing temporal frequency (Δt).

At each velocity, five difficulty levels were presented by exponentially decreasing the signal-to-noise ratio. The signal consisted of dots moving coherently toward a specific direction, and the noise consisted of dots moving randomly. In each level, the signal-to-noise ratio decreased by a factor of 37%: in difficulty level 0 all dots (100%) moved in the same direction and for level 1: 63.1%, level 2: 39.8%, level 3: 25.1%, and level 4: 15.8%. Eight trials with four cardinal and four oblique motion directions were presented at each difficulty level. The participants were tested binocularly in a dimly dark room sitting 60 cm in front of a computer screen. The task was to point out or verbally state the coherent motion direction of the dots on the screen with the guidance of colored arrows. To ensure comprehension of the task all children performed a trial set of ten items before initiation. The scores on the VMCT, indicating the accuracy in global motion perception, ranged from 0 (no correct answer) to a maximum of 1.0 (all answers correct). The three velocity programs on the VMCT were completed by 37 C&Y with CP, functioning at GMFCS I, GMFCS II-III, and 36 TD C&Y, all aged 8–20 years (Figure 1). Five children with CP did not complete the VMCT at all velocities and were excluded from the data analysis, for characteristics of these children see Supplementary Table 2, Additional file 1.

### 2.3 Visual acuity

Binocular visual acuity with habitual correction at 3 meters was evaluated with the KM chart or the Lea symbols chart, based on the participant's age and ability. The best measurable visual acuity is −0.3 logMAR, and binocular visual acuity was categorized as normal/near normal if < 0.5 logMAR, as defined by the World Health Organization.

### 2.4 Raven's colored progressive matrices, Raven's CPM^^

The Raven's CPM was used to give a general estimate of analytical ability (31), hereafter referred to as fluid intelligence. The Raven's CPM is a copyrighted assessment tool owned by Pearson, and permission to use the test was obtained. This study was conducted independently by the researchers, and Pearson has no liability regarding its methodology, results, or conclusions. The task in the Raven's CPM was to complete a pattern by identifying the missing piece in a booklet. The maximal total raw score of 36 indicates fluid intelligence at the level of a typical developing eleven-year-old child. One child could neither complete the Raven's CPM nor the VMCT at all velocities and was therefore excluded.

### 2.5 Cerebral Visual Impairment Questionnaire (CVIQ)

The CVIQ is a parental questionnaire and a structured history-taking inventory capturing features and behaviors of CVI in children, originally developed by Macintyre-Beon and colleagues (15). Results of the CVIQ have shown consistency and reproducibility in a group of TD children, in which few CVI features were reported (15). In the present study, the modified Swedish version of the CVIQ with 39 items, was used (16). Parents rate how frequently their child has difficulties with vision-related activities in daily life on an ordinal scale ranging from 0–4: never (never occurs), rarely (once or twice per month), sometimes (once or twice per week), often (once or twice per day), and always (more often than above). The maximum score is 156 and low scores indicate none/few reported difficulties. An example of a question is: “Does your child have difficulty catching a ball?”. All questions constituting the CVIQ are presented in a study by Hellgren and colleagues (16). The CVIQ was answered by parents to 67 children with CP, functioning at GMFCS I-IV, and 40 TD children, all aged 6–18 years (Figure 1). All questions were answered, except for two children with CP (GMFCS IV), who had twelve and two omitted answers, respectively.

### 2.6 Statistical analyses

Descriptive information about the participants and results from the visual acuity test, the Raven's CPM, the VMCT, and the CVIQ are presented as numbers, percentiles, percent, mean (SE), and/or median [min, max] values. Non-parametric statistics, the Mann-Whitney U-test, and the Kruskal Wallis test were used to analyze possible differences in CVIQ scores for the groups (CP/TD, and GMFCS I/GMFCS II-III/TD). A Pearson Chi-Square test was used to compare proportions between the groups.

Two different Generalized Estimating Equation (GEE) models were applied to analyze global motion perception accuracy, as measured with the VMCT. The first model compared global motion accuracy between the TD and CP groups, with the VMCT score as the dependent variable. Group (TD and CP) was the between-subject factor, Difficulty level (0–4) and Velocity (low, medium, high) were within-subject factors, and the Covariates were fluid intelligence as measured with the Raven's CPM raw scores, and age. The second model examined the effect of GMFCS levels (I, II-III, and TD) on VMCT scores across velocities and difficulty levels, with the same dependent variable, within-subject factors, and covariates as in the first model.

Spearman's correlation coefficient (Rho_s_) was used to describe possible associations between VMCT scores and the CVIQ scores for the whole sample. All statistical analyses were performed with SPSS (version 27 and 28, SPSS Inc., Chicago, IL, USA). The significance level was *p* < 0.05.

## 3 Results

### 3.1 Descriptives of the participants

Age and sex did not differ significantly between the groups (CP/TD, and GMFCS I/GMFCS II-III/TD) who performed the VMCT (Figure 1). The participants (*n* = 73) had a normal/near-normal corrected binocular visual acuity (< 0.5 logMAR). On the Raven's CPM the CP-group (*n* = 37) had a lower raw median [min, max] score than the TD-group (*n* = 36); 33 (12, 32) vs. 35 (32, 33), (*U* = 400.50, *p* = 0.003). There was no difference in Raven's CPM raw scores between the GMFCS I group and the GMFCS II-III group (*U* = 107.50, *p* = 0.519).

### 3.2 Global motion perception

#### 3.2.1 Comparisons between the CP-group and the TD-group

Results from the first GEE model indicated a vulnerability in global motion perception in the CP-group compared to the TD-group, global mean (SE) scores on the VMCT; 0.64 (0.03) vs. 0.70 (0.01), (Wald = 4.06, *p* = 0.04). At high and low velocities, the CP-group displayed a higher disparity in scores from the TD-group than at a medium velocity, see Figure 3a (the interaction Group by Velocity was significant; Wald = 8.98, *p* = 0.011).

**Figure 3:**
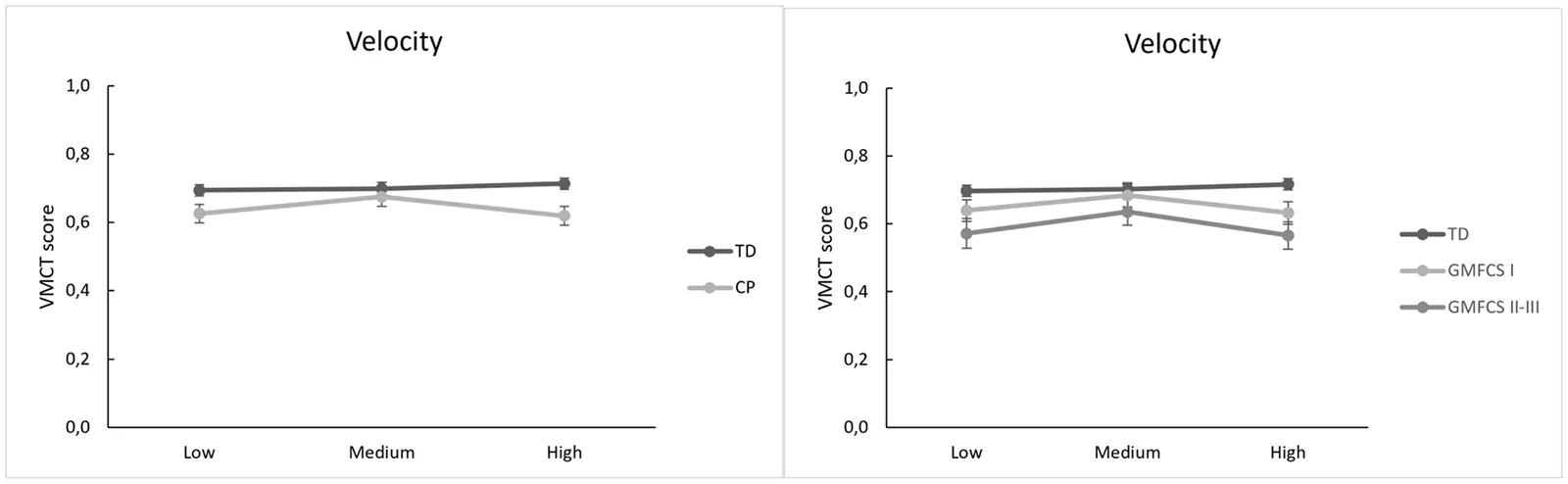
This figure illustrates Visual Motion Coherence Test (VMCT) mean scores (2SE) for each velocity: low, medium, and high, according to groups; **(a)** participants typically developing (TD) and with cerebral palsy (CP), and **(b)** Gross Motor Function Classification System, GMFCS I, GMFCS II-III and TD. The maximum score on the VMCT is 1.0 and low scores indicate global motion perception deficits.

Difficulty level affected both groups such as when the task's difficulty increased (for all velocities combined), scores decreased, more so for the CP-group compared to the TD-group, see Figure 4a, (the interaction effect Group by Difficulty level was significant; Wald = 23.85, *p* < 0.001).

**Figure 4:**
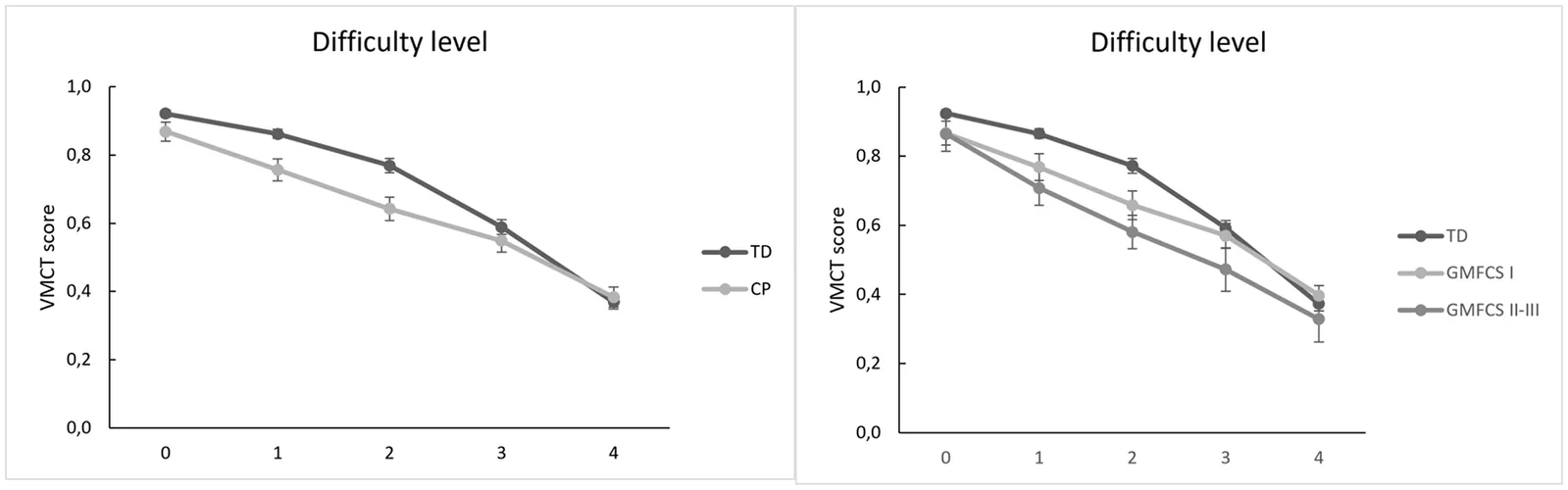
This figure illustrates Visual Motion Coherence Test (VMCT) mean scores (2SE) at increasing difficulty levels, 0–4, for all velocities combined (low, medium, and high), according to groups; **(a)** participants typically developing (TD) and with cerebral palsy (CP), and **(b)** Gross Motor Function Classification System, GMFCS I, GMFCS II-III and TD. The maximum score on the VMCT is 1.0 and low scores indicate global motion perception deficits.

When comparing the results on each velocity by difficulty level, there was no significant difference between the groups, see Figure 5a–c (the interaction Group by Velocity by Difficulty level was not significant; Wald = 14.23, *p* = 0.08).

**Figure 5:**
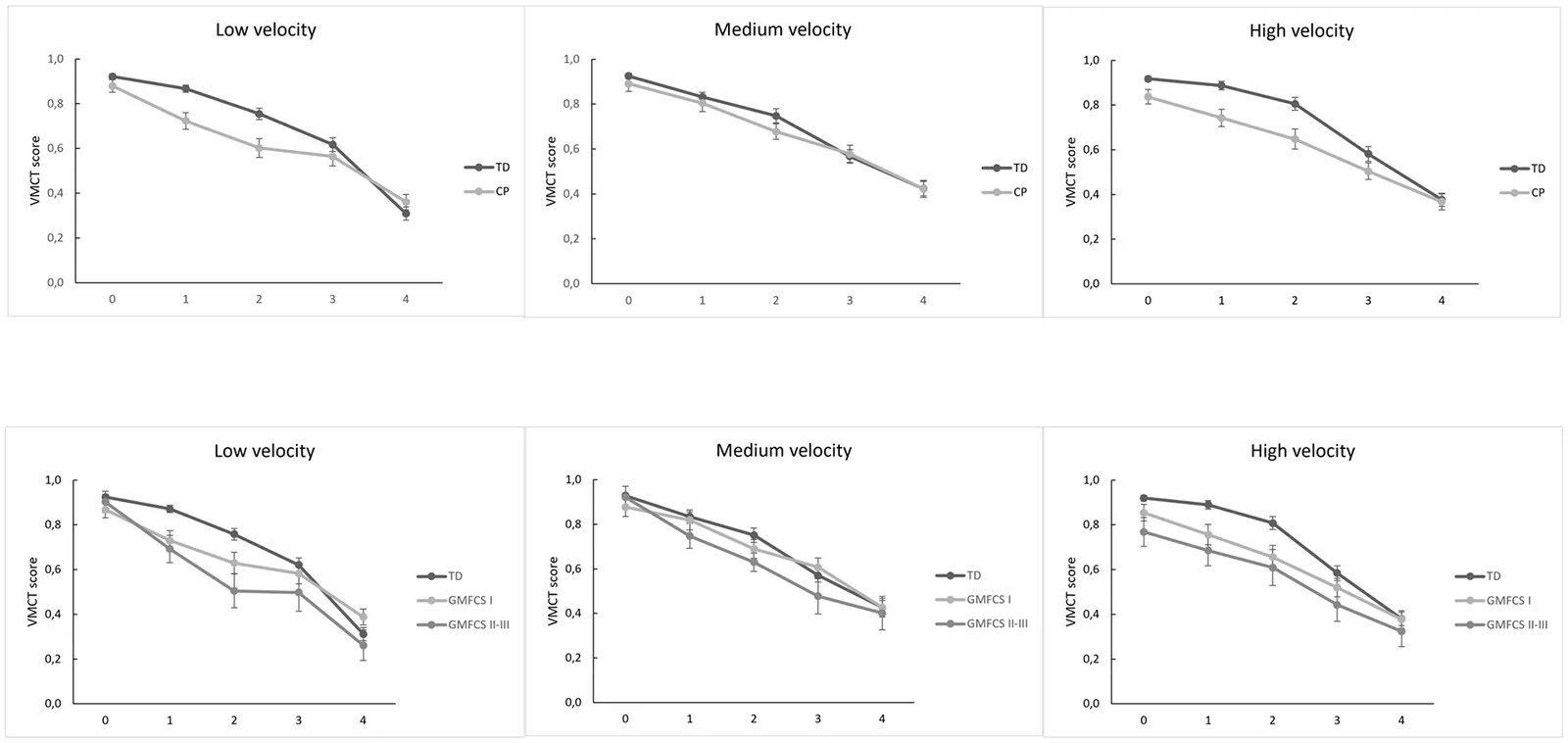
This figure illustrates Visual Motion Coherence Test (VMCT) mean scores (2SE) at increasing difficulty levels, 0–4, for each velocity for the participants typically developing (TD) and those with cerebral palsy (CP) at: **(a)** low, **(b)** medium, and **(c)** high velocity, and for the Gross Motor Functioning Classification System, GMFCS I and GMFCS II-III and TD at **(d)** low, **(e)** medium, and **(f)** high velocity. The maximum score on the VMCT is 1.0 and low scores indicate global motion perception deficits.

For the CP and the TD-group combined (*n* = 73), there was a positive relationship between global motion perception and fluid intelligence, as a high global score on the VMCT was associated with a high raw score on the Raven's CPM (Wald = 60.43, *p* < 0.001, ß = 0.027), while no effect was found for age (Wald = 0.37, *p* = 0.54).

#### 3.2.2 Comparisons between the GMFCS I, the GMFCS II-III, and the TD group

Results from the second GEE model indicated a vulnerability in global motion perception in the GMFCS II-III group compared to the GMFCS I and the TD-group, global mean (SE) scores on the VMCT; 0.59 (0.04), 0.65 (0.03), 0.71 (0.01), (Wald = 8.48, *p* = 0.014). At high and low velocities, the GMFCS II-III group displayed a higher disparity in scores from the other groups (GMFCS I and TD), see Figure 3b (the interaction Group by Velocity was significant; Wald = 14.55, *p* = 0.006). As the difficulty level of the task increased, the performance of the GMFCS II-III group declined more compared to the other groups, see Figure 4b (the interaction Group by Difficulty level was significant; Wald = 30.23, *p* < 0.001). For the GMFCS II-III group, the scores on the VMCT decreased more rapidly by increasing difficulty when the motion stimuli were presented at high and low velocities compared to the other groups, see Figure 5d–f (the interaction Group by Velocity by Difficulty level was significant; Wald = 46.01, *p* < 0.001).

### 3.3 Reported features of Cerebral Visual Impairments

The CP-group had more reported difficulties with vision-related activities in daily life than the TD-group, median [min, max] CVIQ score; 28.0 [0,132], vs. 4.5 [0, 58], (*U* = 2,415.00, *p* < 0.001). Fifty-eight percent (*n* = 39/67) of the children with CP had CVIQ scores above 23.90, the 90th percentile of the TD-group (Figure 6a).

**Figure 6:**
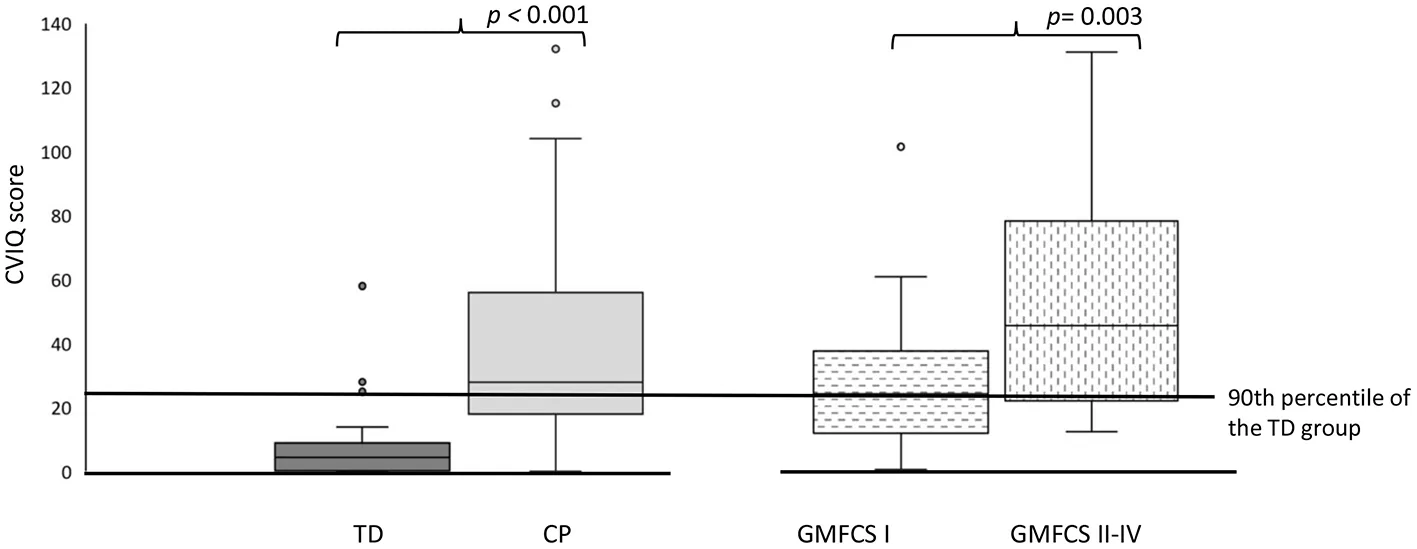
This figure illustrates Cerebral Visual Impairments Questionnaire (CVIQ) median scores for the **(a)** participants typically developing (TD) and those with cerebral palsy (CP), and **(b)** Gross Motor Function Classification System I-group and GMFCS II-IV-group. The 90th percentile of the TD group, 23.90, is indicated with a line. The box represents the interquartile range (i.e., the first and third quartile or 25th to 75th percentile) and the line inside the box shows the median. The whiskers represent the minimum and the maximum values not including the outliers. The circles outside the whiskers represent the outliers. The maximum score on the CVIQ is 156 and high scores indicate CVI.

The GMFCS II-IV group had more reported difficulties with vision-related activities in daily life than the GMFCS I group, median [min, max] CVIQ score; 45.5 [12, 132] vs. 24 [0, 102] (*U* = 793.50, *p* = 0.003) (Figure 6b).

### 3.4 Association between CVIQ score and VMCT score

A high score on the CVIQ was associated with a low score on the VMCT for the CP and the TD-group combined (*n* = 69). There was a negative correlation between the CVIQ scores and the VMCT scores; global mean: Rho_s_ −0.41, *p* = 0.001, and at low, medium, and high velocity (Rho_s_ = −0.44; *p* < 0.001, Rho_s_ = −0.27; *p* = 0.03, Rho_s_ = −0.44; *p* < 0.001).

## 4 Discussion

In this study, visual perception was explored in a controlled environment with a computer-based test and in daily life using parental reports. Consistent with our hypotheses, a vulnerability in global motion perception was detected in the C&Y with CP, most prominent in the group requiring assistance to walk, i.e., functioning at higher GMFCS levels. In addition, more than 50% of the C&Y with CP were reported to have difficulties with vision-related activities, indicating CVI.

To our knowledge, this is the first study to investigate global motion perception with a focus on velocity in individuals with CP. At low and high velocities, we found a reduced capacity to perceive directions of visual motion in the CP-group, more so at GMFCS II-III, compared to the TD-group. At the medium velocity, the performance was more similar between the groups. The reduced performance at the low velocity for the CP-group aligns with the findings of a study indicating impaired global motion perception at a low velocity in children between five to 15 years of age with verified CVI (33). At lower velocities, previous studies have demonstrated that global motion perception matures later compared to higher velocities in TD children (34, 35). Even though we controlled for age in the statistical model, maturation may have impacted the performance of the C&Y with CP. Maturation relates somehow to experience, and in the present study, the children with CP can be expected to have less visual perceptual experience due to their motor disorder. Sensory and perceptual disturbances may both be an accompanying disorder and attributed to the secondary consequences of the activity limitations in individuals with CP (4).

A sensitivity to fast-moving objects could be a possible explanation for the reduced motion perception at the high velocity for the CP-group. In a previous study, fast-moving objects and/or fast movements were described to elicit behaviors such as frequent eye-blinking and/or averted eye gaze, in children with spastic diplegic CP with a perceptual disorder (22). In the present study, the number of children with bilateral/diplegic spastic CP were overrepresented in the GMFCS group II-III, including all except one. A behavior of turning the gaze away from the stimuli would negatively affect the performance (22).

In the present study, scores on the CVIQ above the 90th percentile of the TD-group were reported by most of the parents. This indicates that difficulties with vision-related activities affect many, but not all, C&Y with spastic CP in daily life. Associations between results on the CVIQ and the VMCT imply that the CVIQ could be promising in identifying deficits in global motion perception. Results are congruent with the findings of a population-based study on children born extremely prematurely, in which many reported features of CVI on the CVIQ were supported fairly well by a low result on the VMCT, indicating global motion perception deficits (16). The CVIQ was not specifically developed for children with movement disorders, and some items are related to walking ability. Consequently, the parents of the two children functioning at GMFCS IV could not answer all the questions. Nevertheless, despite not obtaining any scores on the non-responding items, these children still had a high total score on the CVIQ. The CVIQ provides a detailed description of children's vision-related difficulties in daily life, is easy to administer, and can be used as a complement to other assessments, also outside the clinic.

Overall, results from the present study confirm that visual perception needs to be considered when evaluating the functioning of an individual with CP (4, 5, 11). Motor behavior is regulated by a comprehensive sensorimotor network (1) and common walking difficulties in CP, such as crouch gait (36), foot drag, and frequent falls (32) might be overattributed to musculoskeletal impairments (37).

### 4.1 Study strengths and limitations

A methodological strength of this study is that the properties of the three velocity programs used for the VMCT are well described. The velocity of visual motion stimuli is generated by the spatial amplitude and the temporal frequency. In this study, the spatial amplitude, which is more sensitive to maturation (38), was kept constant while the temporal frequency was changed. This facilitates control over the test results.

Consistent with findings in other populations, we found that low performance on the Raven's CPM, measuring fluid intelligence, was associated with difficulties with global motion perception and vison-related difficulties in daily life (16, 30). We minimized the influence of fluid intelligence and age on the VMCT results by including them as covariates in the statistical model. One could argue that the test used for measuring fluid intelligence, the Raven's CPM, poses challenges to visual perception. However, the Raven's assesses the ability to interpret stationary visual patterns whereas the VMCT assesses the ability to perceive visual motion, consequently, they measure different aspects. An additional strength of this study is that all tests of corrected visual acuity revealed normal/near normal visual acuity. Consequently, we can infer that our results from the VMCT were not significantly influenced by variations in visual acuity, age, and fluid intelligence.

A limitation of this study is that many of the children functioning at higher GMFCS levels (GMFCS III and IV) with reported data on the CVIQ declined to participate in examinations, possibly due to the outbreak of COVID-19 during data collection. Consequently, the participants with CP were fairly high-functioning and generalizations of the results should be made with caution. Another potential limitation of this study is that reduced attention span may have influenced some participants. The computer-based testing covered numerous trials (120 trials), and despite short breaks, the testing was attentionally demanding. Integrating visual, cognitive, and attentional systems to inhibit visual noise might be especially challenging for children with CP, more so for those functioning at higher GMFCS levels with an increased prevalence of cognitive impairments (39). Nevertheless, visual perceptual dysfunction is subject to fatigue, and reduced visual attention is a feature of CVI (40).

In this study, low velocity on the VMCT was set to 0.7 deg/sec, whereas high velocity was set to 2.2 deg/sec. In previous research exploring global motion perception in various populations, the properties of the stimuli have been manipulated in different ways (38) and low velocity has been described both as 1 deg/sec (34) and as 3.5 deg/sec (41). The use of varied methodologies and limited research on individuals with CP make it challenging to compare test results across studies.

## 5 Conclusion

The velocity-dependent vulnerability in global motion perception and the reported features of CVI in C&Y with spastic CP is important to consider when evaluating functioning. In this study, findings indicate that C&Y with greater limitations in motor functioning (GMFCS II-IV) have more difficulties with visual perception. Children at high GMFCS levels can be expected to have less experience in exploring the environment due to their motor difficulties, and this underscores the importance of promoting motor and perceptual experiences. For future perspectives, it would be useful to develop a screening version of the Swedish CVIQ, considering the GMFCS levels, for detecting CVI in children with CP. Moreover, it would be of value to explore if visual adaptations during motion-related activities, such as walking and moving in dynamic environments, facilitate functioning.

## Data Availability

The raw data supporting the conclusions of this article will be made available by the authors, without undue reservation.
