# Supplementary material for: Motion matters: visual perception and motor functioning in children and youths with spastic cerebral palsy
Source: Front Neurol. 2026 Sep 25;17:1921002. doi: 10.3389/fneur.2026.1921002 (PMC13649431; doi:10.3389/fneur.2026.1921002)
Supplement: Supplementary file 1 [file Table_1.docx]

Additional File 1.

Supplementary Table 1. Descriptives for the typically developing (TD) children and those with cerebral palsy (CP) who declined to participate in visual tests. Results from the Cerebral Visual Impairment Questionnaire (CVIQ) are presented.

|  | TD children declining visual tests  *n*= 6 | Children with CP declining visual tests  *n*= 29 |
| --- | --- | --- |
| Age, median [min, max] years: months | 10:1 [8:0, 12:4 ] | 11:4 [6:3, 17:9] |
| Sex, male/female, n | 5/1 | 19/10 |
| Subtype, unilateral/bilateral, n | na | 7/22 |
| GMFCS level, n | na | I: 9, II: 10, III: 8, IV: 2 |
| Born prematurely, n | na | 14 |
| CVIQ raw score, median [min, max] | 2.5 [0, 25] | 28 [0, 132] |

Supplementary Table 2. Descriptives and results for the children with cerebral palsy (CP) not performing at all three velocities at the Visual Motion Coherence Test (VMCT). Results from the visual acuity test, Cerebral Visual Impairment Questionnaire (CVIQ), and Raven´s Coloured Progressive Matrices (Raven´s CPM) are presented.

|  | Children with CP not performing at all velocities of the VMCT, *n*= 5 |
| --- | --- |
| Age, median [min, max] years: months | 11:6 [7:0, 12:2] |
| Sex, boys/girls, n | 5/0 |
| Subtype, unilateral/bilateral, n | 2/3 |
| GMFCS level, n | I: 2, II: 2, III: 0, IV: 1 |
| Born prematurely, n | 4 |
| ADHD or/and autism, n | 1 |
| Binocular visual acuity, median [min, max], LogMAR | 0.00 [0.00, 0.22] |
| CVIQ median [min, max] score (*n*=3) | 42 [24, 61] |
| Raven´s CPM, raw score median [min, max] (*n*=4) | 19 [17, 32] |
